# A review of sources of resistance to turnip yellows virus (TuYV) in *Brassica* species

**DOI:** 10.1111/aab.12842

**Published:** 2023-08-15

**Authors:** Kyle Macleod, Shannon F. Greer, Lawrence E. Bramham, Ricardo J. G. Pimenta, Charlotte F. Nellist, Dieter Hackenburg, Graham R. Teakle, Guy C. Barker, John A. Walsh

**Affiliations:** ^1^ School of Life Sciences University of Warwick Warwick UK; ^2^ Imperial College London UK; ^3^ Rothamsted Research, West Common Harpenden UK; ^4^ NIAB Cambridge Crop Research Cambridge UK; ^5^ KWS Einbeck Germany

**Keywords:** brassica, QTL, quantitative resistance, turnip yellows virus, *TuYR*, TuYV

## Abstract

Turnip yellows virus (TuYV; previously known as beet western yellows virus) causes major diseases of *Brassica* species worldwide resulting in severe yield‐losses in arable and vegetable crops. It has also been shown to reduce the quality of vegetables, particularly cabbage where it causes tip burn. Incidences of 100% have been recorded in commercial crops of winter oilseed rape (*Brassica napus*) and vegetable crops (particularly *Brassica oleracea*) in Europe. This review summarises the known sources of resistance to TuYV in *B. napus* (AACC genome), *Brassica rapa* (AA genome) and *B. oleracea* (CC genome). It also proposes names for the quantitative trait loci (QTLs) responsible for the resistances, ***Tu**rnip **Y**ellows virus **R**esistance* (*TuYR*), that have been mapped to at least the chromosome level in the different *Brassica* species. There is currently only one known source of resistance deployed commercially (*TuYR1*). This resistance is said to have originated in *B. rapa* and was introgressed into the A genome of oilseed rape via hybridisation with *B. oleracea* to produce allotetraploid (AACC) plants that were then backcrossed into oilseed rape. It has been utilised in the majority of known TuYV‐resistant oilseed rape varieties. This has placed significant selection pressure for resistance‐breaking mutations arising in TuYV. Further QTLs for resistance to TuYV (*TuYR2*‐*TuYR9*) have been mapped in the genomes of *B. napus*, *B. rapa* and *B. oleracea* and are described here. QTLs from the latter two species have been introgressed into allotetraploid plants, providing for the first time, combined resistance from both the A and the C genomes for deployment in oilseed rape. Introgression of these new resistances into commercial oilseed rape and vegetable brassicas can be accelerated using the molecular markers that have been developed. The deployment of these resistances should lessen selection pressure for resistance‐breaking isolates of TuYV and thereby prolong the effectiveness of each other and extant resistance.

## Graphical abstract

Turnip yellow virus (TuYV) is an important/damaging pathogen for brassicas, including oilseed rape (*Brassica napus*; A+C genomes). Annual UK losses of 67 million GBP have been estimated in oilseed rape; losses in mainland Europe must be much greater. Many resistant commercial varieties are available, however they are all based on the same resistance source (*TuYR1* from the A‐genome). New sources of resistance combined from both A‐ and C‐genomes are available in resynthesised *B. napus* lines (e.g., by combining *TuYR7*+*TuYR8*).

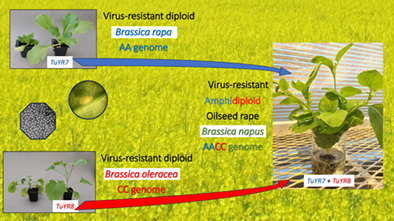

## 1 INTRODUCTION

Turnip yellows virus (TuYV), formerly known as a European isolate of beet western yellows virus (BWYV) unable to infect sugar beet (Graichen & Rabenstein, 1996), is a polerovirus from the family *Solemoviridae* (formerly *Luteoviridae*). It is a non‐segmented positive‐sense single‐stranded RNA virus (+ssRNA). Its genome has seven open reading frames (ORFs), ORF0 is a suppressor of host gene silencing, ORF1 is a genome linked viral protein (VPg) (Reinbold et al., 2013), ORF1/2 (encoded through a −1 ribosomal frameshift) is an RNA dependent RNA polymerase, ORF3a and ORF4 are movement proteins, ORF3 and ORF5 are the major and minor coat proteins, respectively that have been implicated in vector specificity and transmission efficiency (Brault et al., 2005; Schiltz et al., 2022; Smirnova et al., 2015).

TuYV is transmitted in a non‐persistent manner by aphid vectors (Schliephake et al., 2000), with *Myzus persicae* being the main vector in Europe (Asare‐Bediako et al., 2020). Aphids acquire the phloem‐limited virus while feeding on infected plant sap, retain it for life and spread it through subsequent feeding on uninfected plants.

The symptoms of TuYV infection can often go undiagnosed as they are indistinguishable from abiotic stress, notably the yellowing and reddening of leaves and stunted growth associated with nutrient stress (Newbert, 2016).The virus has a wide host range, being able to infect many crop and weed species (Stevens et al., 2008). This is an important factor in TuYV epidemics, as crop‐rotations can do little to lessen TuYV abundance as weeds and winter crops provide overwintering reservoirs for future TuYV spread. TuYV causes significant yield losses in brassica crops, with infection shown to reduce the seed yield of oilseed rape (OSR; canola; *Brassica napus*) plants by 40%–50% (Schröder, 1994) and cause yield losses in OSR crops of 11%–46% (Congdon et al., 2020; Graichen & Schliephake, 1999; Jay et al., 1999; Jones et al., 2007). It has been reported to reduce OSR yield by as much as 30% in the UK, equating to in excess of £67 million a year (Nicholls, 2013). TuYV has been found to cause significant weight yield reductions in cabbage (16%–36%) (Hunter et al., 2002) and Brussels sprouts (30%–65%) in the UK (Walsh et al., 2011). Infection can also cause tip burn in cabbage, resulting in unmarketable produce (Hunter et al., 2002).

Brassica crops are an important source of oilseed and vegetables globally. In 2019, there was an estimated 10.5 million tonnes production of rapeseed oil in Europe, representing 43% of global production (FAO, 2019). The estimated value of global imports and exports of rapeseed oil in 2020 totalled USD7.7 billion and USD7.5 billion, respectively (FAO, 2020c). The global estimate of cabbage production was 70.8 million tonnes, with 9.6 million tonnes in Europe (FAO, 2020a). The estimated value of global imports and exports of cabbage in 2020 was USD2.3 billion and USD1.9 billion, respectively (FAO, 2020b).

**FIGURE 1:**
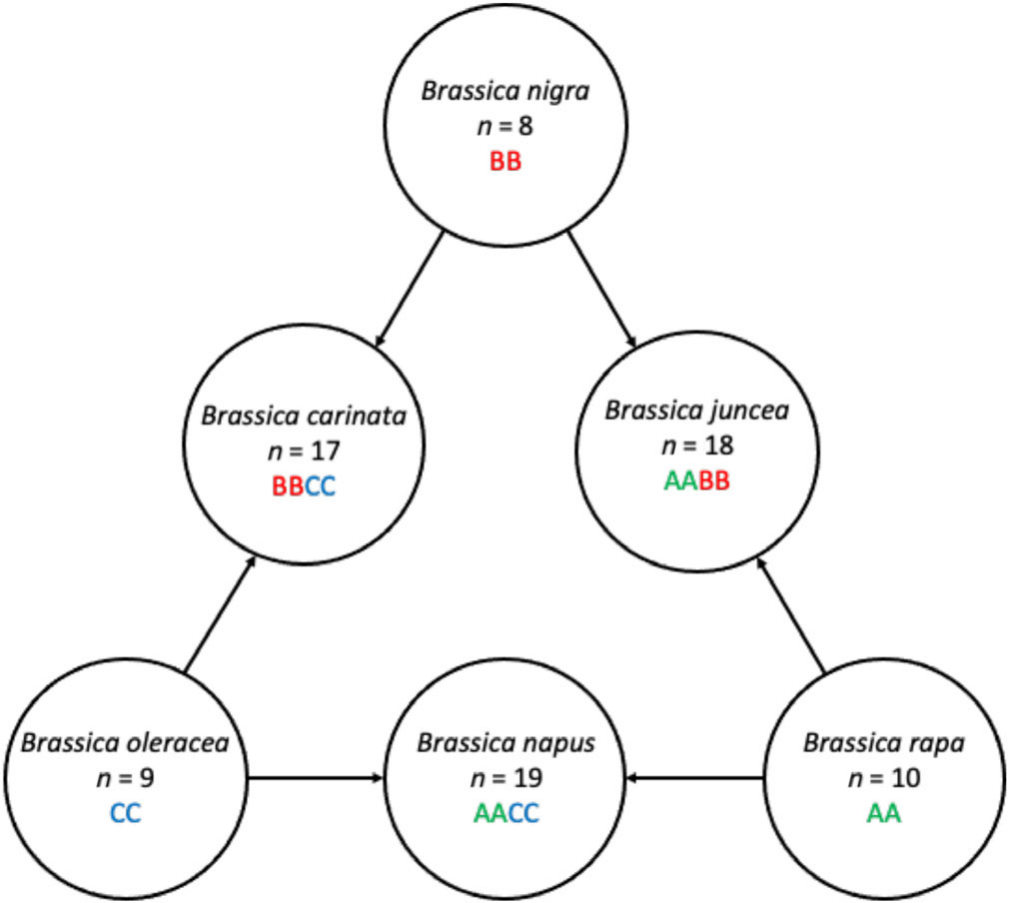
Triangle of Nagaharu showing the relationships between the six major brassica crop species. *Brassica nigra*, *Brassica oleracea* and *Brassica rapa* are diploids, while *Brassica carinata*, *Brassica juncea* and *Brassica napus* are allotetraploids derived from each pair of the three diploid species. *Source*: Adapted from Nagaharu (1935).

The genome triplication in a brassica ancestor (Cheng et al., 2014) and subsequent speciation has driven diversification within the *Brassica* genus. The six major species within the genus comprise three diploid species (*Brassica rapa*, *Brassica nigra*, and *Brassica oleracea*) and three allotetraploids (*Brassica juncea*, *B. napus*, *Brassica carinata*) derived from each pair of the three diploid species (Figure 1). This interesting relationship between species allows for the exchange of genetic elements between the diploid species and introduction of new genetic elements into the allotetraploid brassica species through the hybridisation of diploid species to create resynthesised allotetraploids.

As of 2018, there was an EU‐wide ban for outdoor use of three neonicotinoids, the main systemic insecticide used against aphids. Without such control of the vector a resulting surge of disease carrying aphid populations can be expected. For example, the increase of virus yellows complex in 2020 resulted in the emergency use of neonicotinoids for the sugar beet industry in the UK (Dewar & Qi, 2021). Alternative strategies are needed to control both vector and virus, with a paradigm shift away from ecologically harmful chemicals to which *M. persicae* has developed resistances (Anstead et al., 2005; Bass et al., 2014), towards a more durable and sustainable approach, such as breeding for genetic resistance.

The first genetic resistance to TuYV was mapped in the resynthesised *B. napus* line ‘R54’ and has been incorporated into many TuYV‐resistant varieties across Europe. Nevertheless, utilising numerous sources of resistance to TuYV offers a more robust and durable approach to tackling the TuYV epidemic. New sources of resistance should reduce the likelihood of resistance‐breaking mutations arising in the TuYV population.

In this article, we review the known sources of TuYV resistance in brassica from our own research and that of others. We also propose names for the resistance quantitative trait loci (QTLs) (***Tu**rnip **Y**ellows virus **R**esistance*, *TuYR*) in the different brassica species (Table 1).

**TABLE 1:** Sources of resistance to turnip yellows virus in *Brassica napus*, *Brassica rapa* and *Brassica oleracea*.

Gene^a^	Species	Subgroup	Accession/cultivar	Chr^b^	Position (cM)^c^	Inheritance	Variation explained (%)	Flanking markers	Reference
*TuYR1*	*B. napus*	resynthesised	R54	A04	16.0 or 17.0	Dominant	22.2–52.7	STS3‐e32m50‐447‐320 and STS1‐e31m48‐437	Dreyer et al., 2001; Juergens et al., 2010
*TuYR2*	*B. napus*	*oleifera*	Yudal	A04	10.0, 12.9 or 15.5	Partially dominant	18.2–36.0	scaffoldv4_71_1383505 and scaffoldv4_649_37158	Hackenberg et al., 2020
*TuYR3*	*B. rapa*	–^d^	ABA15005	A02	45.9	Partially dominant	23.1	Bn‐A02‐p6627382 and Bn‐A02‐p13123802	Greer et al., 2021
*TuYR4*	*B. rapa*	–	ABA15005	A06	78.0	Partially dominant	17.2	Bn‐A06‐p18144127 and Bn‐A06‐p24156940	
*TuYR5*	*B. oleracea*	–	JWBo12	C05	47.3	Partially dominant	10.6	Bn‐scaff_18181_1‐p620712 and Bn‐scaff_23186_1‐p18537	
*TuYR6*	*B. oleracea*	–	JWBo12	C05	81.4	Partially dominant	11.5	Bn‐scaff_18181_1‐p620712 and Bn‐scaff_23186_1‐p18537	
*TuYR7*	*B. rapa*	–	ABA15001	A04	–	Dominant	22.0	–	Unpublished
*TuYR8*	*B. oleracea*	–	JWBo3	–	–	Dominant	35.3	–	Unpublished
*TuYR9*	*B. oleracea*	–	JWBo1	C07	–	Dominant	–	–	Unpublished
–	*B. napus*	*oleifera*	Liraspa‐A	–	–	–	–	–	Congdon et al., 2021
–	*B. napus*	*oleifera*	SWU Chinese 5	–	–	–	–	–	
–	*B. napus*	*oleifera*	SWU Chinese 3	–	–	–	–	–	
–	*B. oleracea*	*botrytis*	Quickheart	–	–	–	–	–	
–	*B. oleracea*	*gemmifera*	Evesham Special	–	–	–	–	–	
–	*B. oleracea*	*capitata*	Savoy King F1	–	–	–	–	–	
–	*B. oleracea*	*italica*	Purple sprouting extra early	–	–	–	–	–	

^*a*^ ***T**urnip **Y**ellows virus **R**esistance* (*TuYR*); resistance genes are named where they are mapped to at least the chromosome level.

^b^ Chromosome; ‘A’ represents the A‐genome of *B. rapa* and ‘C’ represents the C‐genome of *B. oleracea*.

^c^ Where several locations are given, this relates to multiple mapping experiments.

^d^ No data available.

## 2 FIRST REPORT OF RESISTANCE TO TuYV IN *BRASSICA*

The first report of resistance to TuYV was in the resynthesised *B. napus* line R54 (Graichen, 1994), developed at the University of Göttingen, Germany. R54 was generated through the interspecific hybridisation of white cabbage (*B. oleracea* var. *capitata* f. *alba*) and Chinese cabbage (*B. rapa* ssp. *pekinensis*), with the Chinese cabbage being the donor of resistance (Graichen, 1994; Paetsch et al., 2003).

QTL mapping experiments were conducted on a doubled‐haploid (DH) population produced from an F_1_ (derived from a cross between the *B. napus* variety ‘Express’ and the resynthesised line R54) (Dreyer et al., 2001). The progeny of 235 individuals produced from microspore culture were artificially challenged with TuYV using viruliferous aphids, and viral titre was measured with double antibody sandwich enzyme‐linked immunosorbent assay (DAS‐ELISA) with a polyclonal antiserum against TuYV. The plants were genotyped with amplified fragment length polymorphism (AFLP) markers. A 1:1 distribution of susceptible to resistant plants expected for monogenic trait in a DH population was never observed. Additionally, 64% of the markers displayed a segregation pattern deviant from the expected 1:1 ratio. QTL mapping using 143 AFLP markers resulted in 22 linkage groups, which represented only 15 of the 19 *B. napus* chromosomes. The map was 1141 cM in length, with the interval length between two markers ranging from 3.78 to 35.2 cM. The QTL mapping indicated a single QTL on linkage group MS17 (chromosome A04) closely linked to marker E15_083d and with a support interval of 7 cM. The average phenotype variation explained by the QTL over multiple experiments was 50.7%. We have proposed naming this QTL, *TuYR1*.

To facilitate the introgression of *TuYR1* into elite canola lines, numerous genetic markers closely linked to *TuYR1* were developed. The most closely linked published markers were two sequence‐tagged site (STS) markers (Juergens et al., 2010), STS3‐e32m50‐447‐320 (forward: 5′‐GATCCGTTTGGGTCTTGGTA‐3′; reverse: 5′‐TTGATGTGAAACGCACATTG‐3′) localised 0.91 cM from *TuYR1*, the amplicon size in resistant and susceptible individuals was 344 and 385 bp, respectively. The second marker, STS1‐e31m48‐437 (forward: 5′‐ATCGGACATTGGTCAGGTTC‐3′; reverse: 5′‐CATACCCCACTGGTTCTTGG‐3′), was found to co‐localise with *TuYR1*; resistant and susceptible individuals had amplicon sizes of 437 and 376 bp, respectively. Both markers can be easily distinguished by gel electrophoresis.

Field studies using DH populations over three growing seasons showed a good fit to the 1:1 segregation of resistant to susceptible plants, indicative of pre‐winter monogenic resistance (Juergens et al., 2010). However, the authors noted that resistance is most likely controlled by a single major gene, influenced by additional minor genes and may break down at higher temperatures. Therefore, the resistance is not complete but rather a quantitative reduction of virus titre (Dreyer et al., 2001; Juergens et al., 2010).

There are several TuYV‐resistant commercial varieties, including Caletta from Semundo (now Senova, Cambridge, UK), Amalie, Ambassador, Annalise, Architect, Artemis, Aspire, Aurelia from Limagrain (Saint‐Beauzire, France), Darling, Dazzler, Ludger, Temptation from DSV (Lippstadt, Germany), Allessandro, Feliciano from KWS (Einbeck, Germany), Atora, Dominator from Rapool‐Ring (Isernhagen, Germany), Cadran, Coogan from RAGT (Rodez, France), Addition from Soufflet Seeds (Poznań, Poland), DK Excited, DK Expectation, DK Exposé, DK Excentric from Bayer (Leverkusen, Germany) and DMH440 from Dekalb AgResearch (Dekalb, USA). Many of these lines are known to possess resistance derived from *TuYR1*. Growing many varieties with the same source of TuYV resistance is creating a strong selection pressure for resistance‐breaking mutations arising in TuYV populations. Numerous research groups have been searching for further sources of resistance that can be utilised alongside *TuYR1* to reduce selection pressure for resistance‐breaking isolates of TuYV and prolong the lifespan of TuYV resistances in brassica crops.

## 3 FIRST RESISTANCE TO TuYV FOUND IN A NATURAL ALLOTETRAPLOID *B. NAPUS*

The first report of TuYV resistance in a natural *B. napus* came from Hackenberg et al. (2020), who identified a major QTL in the Korean spring variety Yudal. The authors screened a diverse subset of the *B. napus* gene pool comprising 27 DH or inbred lines of winter OSR, spring OSR, Siberian kale, swede and resynthesized *B. napus*, from different regions of the world. Plants were challenged with TuYV using viruliferous aphids and viral titre was measured by triple antibody sandwich ELISA (TAS‐ELISA), where low absorbance (*A*_405nm_) values were indicative of low viral titre and hence resistance, while high *A*_405nm_ values represented susceptibility. Partial resistance (low viral titre) could only be observed in Yudal while the winter OSR Darmor‐*bzh* line was susceptible (high viral titre), with infected plants having *A*_405nm_ values >3‐fold that of Yudal. Using the Darmor‐*bzh* × Yudal DH mapping population (DYDH) (Delourme et al., 2006) as well as two reciprocal backcross (BC_1_) populations, the major QTL was mapped to chromosome A04 and was inherited in a dominant manner. We have named this QTL *TuYR2*.

**FIGURE 2:**
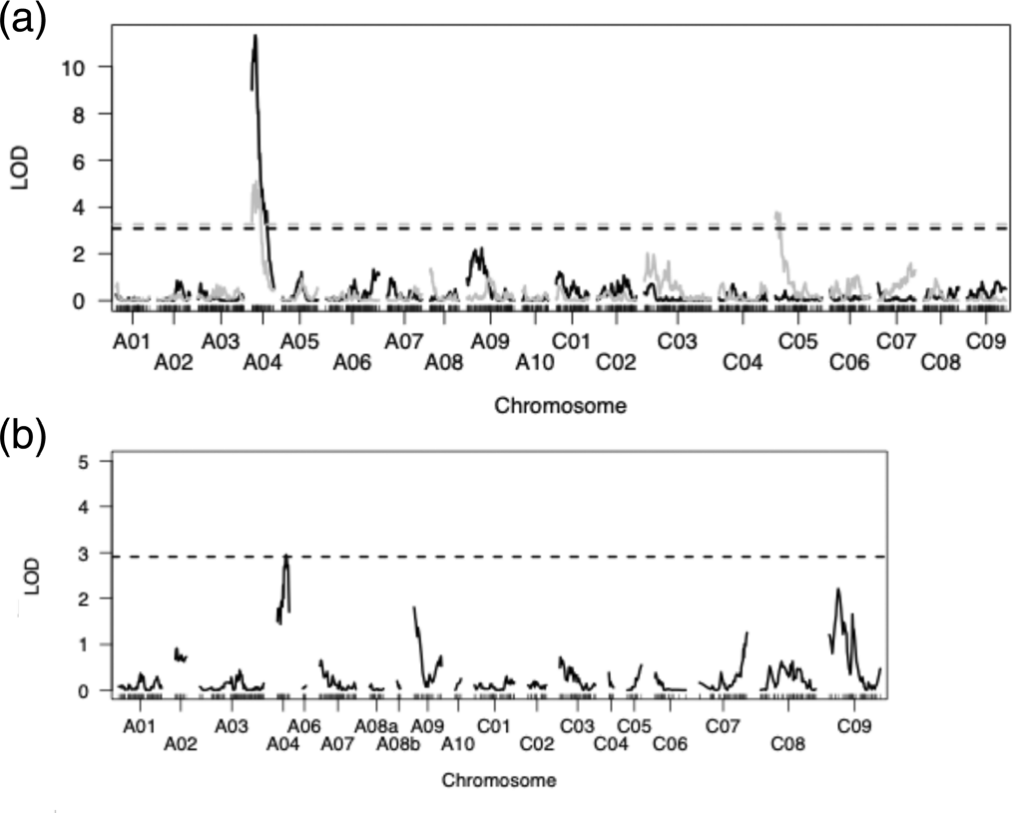
QTL mapping of resistance to turnip yellows virus (TuYV) in *Brassica napus* variety Yudal. (a) Genome wide LOD scores for TuYV‐resistance in two experiments on the Darmoor‐bzh × Yudal doubled‐haploid mapping population (Delourme et al., 2006). The major QTL for resistance to TuYV was found on chromosome A04 in both experiments. This QTL has been named *TuYR2*. The minor QTL on chromosome C05 was found in one of the experiments (grey). (b) Genome wide LOD scores for TuYV‐resistance in a BC_1_ population (Darmor‐bzh × [Yudal × DYDH130]) confirming the presence of *TuYR2* on chromosome A04. Genome wide LOD thresholds (*α* ≤ .05) of 10,000 permutations are indicated as dashed horizontal lines. *Source*: Figure from Hackenberg et al. (2020).

In the first experiment on the DYDH population, the minimal map contained 1298 markers with a linkage map size of 2196.3 cM, a minimum spacing of 1.7 cM and maximum of 13.3 cM. The smallest linkage group was chromosome A04 (65 cM) with 39 markers and the largest was chromosome C03 (196.6 cM) with 114 markers. Non‐parametric QTL analysis detected *TuYR2* on chromosome A04 with a LOD score of 11.3, explaining 36.01% of the phenotypic variation at position 10 cM (Figure 2a). The 1.5 LOD QTL confidence interval spanned 13.9 cM. In the second experiment on the DYDH population, QTL analysis was conducted using parametric analysis and confirmed *TuYR2* on A04 (albeit with a 1.5 LOD interval of 27 cM) as well as identifying a minor QTL on chromosome C05 acting additively without interaction (Figure 2a). Interestingly, the resistance allele for this minor QTL on C05 came from Darmor‐*bzh*. The phenotypic variation explained by *TuYR2* was 18.2%, compared with 13.8% for the minor QTL on C05 (19 cM 1.5 LOD). The differences observed between DYDH experiments could possibly be explained by environmental factors such as differences in sowing dates (autumn and winter), although both experiments were conducted in a glasshouse under similar temperature regimes.

Although two BC_1_ populations were phenotyped for resistance to TuYV, only the one BC_1_ population (Darmor‐*bzh* × [Yudal × DYDH130]), was used for QTL mapping. This population showed a greater number of susceptible individuals and better segregation of TuYV‐partially resistant and TuYV‐susceptible individuals. A subgroup of 107 (out of 200) individuals, representing the range of measured virus titres, were genotyped. Markers polymorphic between parents (*n* = 7790) were assembled into a minimal map containing 567 markers. The overall map size was 934.3 cM, with a minimum spacing of 1.7 cM and maximum spacing of 18.9 cM. The smallest linkage group was chromosome A06 (5.6 cM with 6 markers) and the largest was chromosome A03 (115.8 cM and 79 markers). Mapping confirmed the major QTL, *TuYR2*, on chromosome A04 at position 15.5 cM with an LOD score of 2.95 and explaining 11.9% of the phenotypic variation (Figure 2b). *TuYR2* had a 1.5 LOD interval of 21.3 cM (the entire linkage group).

Physical positions of markers flanking *TuYR2* in the DYDH and BC_1_ populations were determined, by comparison with the *B. napus* genome assembly of Darmor‐*bzh* (GCA_000751015.1). All 1.5 LOD intervals were located on chromosome A04, with the smallest interval of 10.75 Mb (in the first DYDH experiment), 13.79 Mb (in the second DYDH experiment) and 10.79 Mb in the BC_1_ experiment. The smallest interval contained 1262 annotated genes. The interval in Yudal encompassed the smaller R54 *TuYR1* QTL (approximately 2.9 Mb in length), based on the markers in Juergens et al. (2010). As a result of low recombination frequency on chromosome A04 in the BC_1_ population, only 19 informative markers were used representing 21 cM/12.8 Mb of A04 (i.e., 58% of the physical map of A04). Greater resolution was found in the DYDH experiments and enabled mapping of the QTL between markers at positions 0.4 and 12.6 Mb. This highlighted the advantage of homozygous DH populations in mapping the quantitative virus resistance trait in this instance.

Similar to the phenotyping experiments on *TuYR1*, none of the DYDH or BC_1_ populations showed a clear bimodal distribution of susceptible to resistant plants, as would be expected for a 1:1 segregation of a strong monogenic trait. Thus, *TuYR2* may be influenced by additional contributing genes and/or, environmental factors. Alternatively, lack of 1:1 segregation may be an artefact of phenotyping partially dominant traits.

The origin of the *TuYR2* resistance was suggested to be independent of *TuYR1*, as the markers co‐segregating for *TuYR1* (Juergens et al., 2010) were not conserved in Yudal, that is, marker analysis showed that both Yudal and Darmor‐*bzh* possessed the susceptibility alleles.

Interestingly, Congdon et al. (2021) analysed Yudal for resistance to TuYV and stated that it was completely susceptible to TuYV. However, their experimental setup was different to that of Hackenberg et al. (2020). Plants were grown under glasshouse conditions at temperatures ranging from 16 to 23°C, which is different from the 18°C in the latter experiments. Additionally, different isolates of TuYV and different accessions of Yudal were deployed by the two groups.

## 4 THE FIRST EXAMPLE OF RESISTANCE TO TuYV IN THE TWO DIPLOID *BRASSICA* PROGENITOR GENOMES INCORPORATED INTO ALLOTETRAPLOID AACC PLANTS THROUGH RESYNTHESIS

*Brassica napus* (AACC genome) (Figure 1) is considered to have a relatively recent origin (approximately 7500 years ago) and its genetic diversity is limited relative to its diploid progenitor species, *B. rapa* (AA genome) and *B. oleracea* (CC genome) (Chalhoub et al., 2014). These diploid species diverged from one another approximately 3.8 million years ago (Inaba & Nishio, 2002). Extensive breeding programmes to improve oil quality have also further diminished the genetic diversity within *B. napus* (Cowling, 2007; Fu & Gugel, 2010). The *B. napus* genetic bottleneck poses a problem when breeding for traits such as disease resistance. However, it can be overcome by resynthesising *B. napus* through the interspecific hybridisation of the diploid progenitor species and genome duplication to produce allotetraploid plants (AACC) (Greer et al., 2021). Identifying resistance to TuYV in the more diverse diploid brassica genomes of *B. oleracea* and *B. rapa*, and introgressing these into the less diverse *B. napus* through resynthesis and backcrossing is a very attractive strategy for introducing new sources of resistance into OSR.

Greer et al. (2021) found and mapped new sources of TuYV resistance in *B. rapa* (ABA15005) and *B. oleracea* (JWBo12) and proceeded to resynthesise allotetraploid AACC plants (SER19001) through an interspecific cross of the TuYV‐resistant *B. rapa* and *B. oleracea* individuals. This was the first report of a *B. napus* with resistance to TuYV in both the A and C genomes, as well as the first report of a C genome‐associated resistance.

The segregating populations used for mapping showed a continuous broad range of virus titres and not a 1:1 segregation expected for a single completely dominant QTL. This was more indicative of quantitative resistance similar to the *TuYR1* and *TuYR2* resistances. F_1_ populations had virus titres intermediate of the resistant and susceptible lines indicating partially dominant resistance, with the *B. rapa* resistance being less dominant than that in *B. oleracea*.

Mapping experiments for the *B. rapa* resistance were conducted on a segregating BC_2_ population. Genotyping of a subset of 72 *B. rapa* individuals was carried out using the *Brassica* Infinium 60 K array (Illumina, USA); there were 2479 informative markers, of which, 351 represented the overall recombination within the BC_2_ population. The genetic map had a total length of 868.1 cM and comprised 10 linkage groups representative of the 10 *B. rapa* chromosomes. The mean distance between markers was 2.5 cM, with a minimum and maximum distance of 0.6 and 56.2 cM, respectively. The smallest linkage group was chromosome A10 with 3 markers and a length of 1.8 cM, while the largest group was chromosome A09 of 124.3 cM with 46 markers.

**FIGURE 3:**
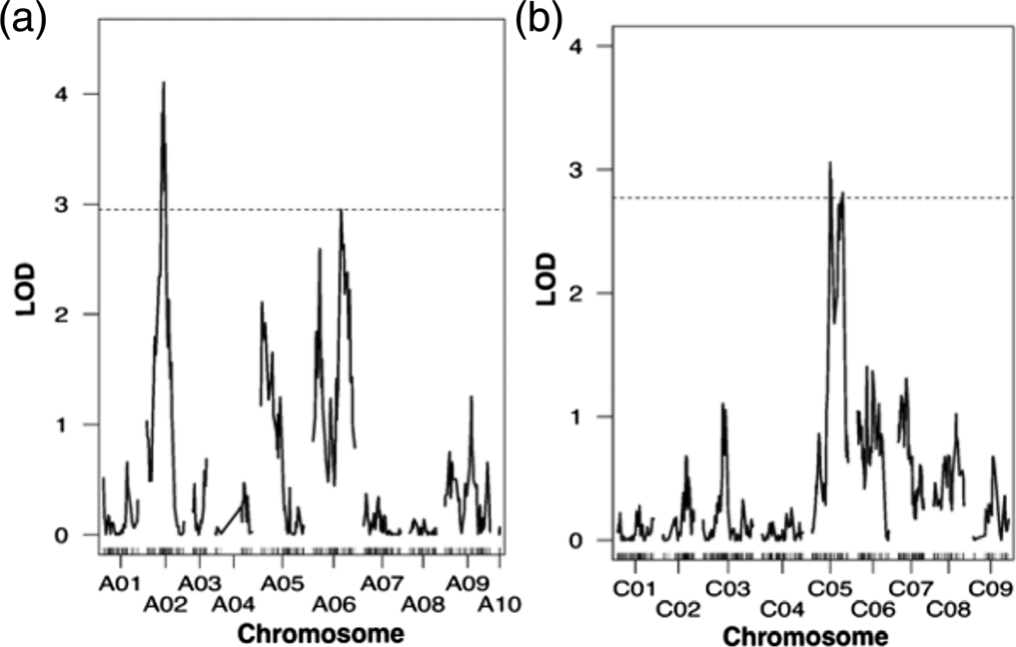
QTL mapping of resistance to turnip yellows virus (TuYV) in *Brassica rapa* and *Brassica oleracea*. (a) A mapping experiment on the *B. rapa* BC_2_ population identified two significant QTLs, one on chromosome A02 (*TuYR3*) explaining 23.1% of the phenotypic variation and another on chromosome A06 (*TuYR4*) explaining 17.2% of the phenotypic variation. (b) A mapping experiment on the BC_1_ *B. oleracea* population identified two distinct QTLs on chromosome C05 at 47.3 cM (*TuYR5*) explaining 10.6% of the phenotypic variation and at 81.4 cM (*TuYR6*) explaining 11.5% of the phenotypic variation. A resynthesised allotetraploid *B. napus* was created through the interspecific hybridisation of a resistant *B. rapa* with a resistant *B. oleracea* and resynthesised plants were confirmed to contain *TuYR3*, *TuYR4*, *TuYR5*, and *TuYR6*. Genome wide LOD thresholds (*α* ≤ .05) of 1000 permutations are indicated as dashed horizontal lines. *Source*: Figure from Greer et al. (2021).

The mapping in the *B. rapa* population identified two significant QTLs on chromosome A02 at 45.9 cM (LOD = 4.10) and A06 at 78.0 cM (LOD = 2.96) which narrowly surpassed the genome‐wide significance threshold of 2.95 (Figure 3a). The QTL on chromosome A02 explained 23.1% of phenotypic variation while the QTL on A06 explained 17.2%. The QTL on A02 had a 1.5 LOD interval of 16 cM, spanning 37.0–53.0 cM, while on A06 the QTL had a 1.5 LOD of 34.5 cM spanning 69.0 to 103.5 cM. We have named the QTL on A02, *TuYR3*, and the QTL on A06, *TuYR4*. Additionally, it was found that *TuYR3* and *TuYR4* interacted positively, while another significant additive model identified *TuYR3* and another non‐interacting QTL on chromosome A06 at 18.6 cM (LOD = 2.56), which did not surpass the genome‐wide significance threshold in the initial one‐dimensional interval mapping. Of the significant major QTLs, *TuYR3* spanned a length of 6.9 Mbp, based on the *B. rapa* Chiifu reference genome (GCA_000309985.3) and contained 1052 genes while *TuYR4* had a length of 4.0 Mbp and contained 795 genes. The authors concluded that the resistance in ABA15005 was controlled by two major QTLs with a smaller additive but non‐interacting QTL on chromosome A06 (18.6 cM) potentially contributing towards the TuYV resistance.

In the *B. oleracea* population, a subset of 115 individuals of a BC_1_ population was genotyped with the *Brassica* Infinium 60 K array (Illumina, USA). In this population, 4409 informative markers were identified and 448 of these were used in the minimal genetic map. The map had a total length of 837.1 cM comprising the nine linkage groups representative of the nine *B. oleracea* chromosomes. The mean distance between two markers was 1.9 cM, with minimum and maximum distances of 0.4 and 27.0 cM, respectively. The smallest linkage group was chromosome C07 (68.4 cM) and the largest was chromosome C03 (131.3 cM). Mapping identified two distinct significant QTLs on chromosome C05 at 47.3 cM (LOD = 3.06) and at 81.4 cM (LOD = 2.81), both surpassing the genome‐wide significance of 2.77 (Figure 3b). However, the 1.5 LOD intervals overlapped, from 41.0 to 88.0 cM, with a length of 47.0 cM and were flanked by the same markers. We have named the QTL at 47.3 cM, *TuYR5* and the QTL at 81.4 cM, *TuYR6*. Based on the *B. oleracea* TO1000 reference genome (GCA_000695525.1), both *TuYR5* and *TuYR6* have a length of 37.0 Mbp and contain 3773 genes. The authors suggest the double peak on C05 was because of an artefact of reduced genotype call rate between the peaks; however, the possibility of two separate QTLs cannot be ruled out. The 1.5 LOD interval of 47.0 cM is quite large, therefore, it should be possible to find recombinants with genomic segments surrounding either peak and use these recombinants to assess the contribution of either QTL.

Greer et al. (2021) successfully rescued embryos from an interspecific cross between the resistant *B. rapa* and *B. oleracea* and after colchicine treatment and selfing, the progeny were confirmed to be allotetraploid AACC. Phenotyping of the resynthesised population (SER19001) showed that the resynthesised line was uniformly resistant, did not show significantly different viral titre from either diploid resistant parent but had a significantly lower titre than that of the susceptible lines used in the diploid mapping. Genotyping confirmed the presence of the resistance‐associated alleles near the peaks of the QTLs *TuYR3*, *TuYR4*, *TuYR5* and *TuYR6*, based on the Bn‐A02‐7840077, Bn‐A06‐18369013, and Bn‐scaff_16082_1‐p278297 markers.

## 5 FURTHER SOURCES OF RESISTANCE FOUND IN THE DIPLOID *B. RAPA* AND *B. OLERACEA*

Screening a range of *B. oleracea* and *B. rapa* lines at the University of Warwick has identified resistance to TuYV in a further *B. rapa* accession and two further *B. oleracea* accessions. A strategy similar to that of Greer et al. (2021) was employed to create a resynthesised allotetraploid *B. napus* from the interspecific cross between the TuYV‐resistant *B. rapa* and one of the TuYV‐resistant *B. oleracea* lines.

In the *B. rapa* experiments, 14 plant lines were tested for resistance to TuYV by quantifying the virus within plant sap using TAS‐ELISA, following challenge with viruliferous aphids. From this initial screen, a *B. rapa* line (ABA15001) showed high levels of resistance to two isolates of TuYV. The TuYV‐resistant line was crossed with the TuYV‐susceptible *B. rapa* ssp. *trilocularis* R‐o‐18; the F_1_ progeny had ELISA values similar to that of the resistant parent, suggesting the resistance is inherited in a dominant manner. Mapping of the resistance was conducted on a segregating BC_1_ population and 48 plants, representing the range of viral titres, were genotyped with the *Brassica* Infinium 60 K array (Illumina, USA). The results indicated a major QTL on chromosome A04, explaining 22% of the phenotypic variation with a 1.5 LOD interval of 15 cM. Further experiments are being conducted on a BC_2_ population in order to fine‐map the QTL interval, potentially down to gene level, to create genetic markers tightly linked with the resistance. We have named this QTL *TuYR7*.

Two *B. oleracea* accessions (JWBo3 and JWBo1) were found to be resistant to TuYV in multiple experiments. For one of the *B. oleracea* accessions, a cross was made with a susceptible line DHSL150 and the F_1_ progeny displayed resistance to TuYV similar to the parental line, suggesting that the resistance is inherited in a dominant manner. A segregating BC_1_ population was used for mapping experiments, where 40 plants were genotyped with the *Brassica* Infinium 60 K array and 200 individuals were genotyped with 30 KASP markers spread over chromosomes C02, C07 and C08. The mapping results indicated a major QTL explaining 35.3% of the phenotypic variation with an interval of 12.5 cM. Further work is underway to reduce the interval and fine map the gene/s responsible in this accession. We have named this QTL *TuYR8*.

In order to deploy these two sources of resistance in arable brassicas, resynthesised allotetraploid AACC plants were obtained by embryo‐rescue following interspecific hybridisation between the TuYV‐resistant *B. rapa* (ABA15001) and *B. oleracea* (JWBo3) lines. The refined genetic markers generated from experiments with the diploid progenitors will be tested against DH resynthesised allotetraploid AACC lines to ensure tight linkage with resistance. Additionally, allotetraploid DH lines with both sources of resistance (*TuYR7* and *TuYR8*), either resistance QTL (*TuYR7* or *TuYR8*) and neither resistance QTL (*tuyr7* nor *tuyr8*) will be challenged with TuYV and compared to assess the contribution of each resistance QTL to the overall level of resistance in the diploid progenitor plants with the *TuYR7* or *TuYR8* genotype. The plant lines possessing the combined *TuYR7* resistance in the A genome and *TuYR8* resistance in the C genome should reduce the selection pressure for resistance‐breaking virus mutations and hence provide more durable resistance than plant lines with just the one source of resistance.

Another *B. oleracea* accession, JWBo1, was found to contain a dominantly inherited QTL on chromosome C07 in both BC_1_ and BC_2_ populations. Further work is underway to investigate candidate genes within the QTL. We propose to name this QTL *TuYR9*.

A large study, involving the screening of 55 *B. napus*, 25 *B. oleracea* and eight *B. rapa* accessions for resistance to TuYV under glasshouse conditions (Congdon et al., 2021) has potentially identified new resistance sources. Three *B. napus* lines and four *B. oleracea* lines were identified as resistant to TuYV (Table 1). However, attempts to map the resistance in these lines was out of the scope of the published work. Hopefully, work is under way to map these sources of resistance. The authors investigated the effect of temperature on the level of resistance in the lines and found an almost total breakdown of resistance at 30°C, except in *B. napus* ‘SWU Chinese 5’, highlighting the potential durability of this resistance in the face of increasing global temperatures.

## 6 CONCLUSIONS

Since the first report of resistance to TuYV in the resynthesised *B. napus* line R54 (Graichen, 1994) and later QTL mapping (Dreyer et al., 2001), there has been a considerable amount of time (19 years) before report of further sources of resistance. However, there are now numerous sources of resistance to TuYV identified in diverse brassica species (Table 1), with or without associated genetic markers. All resistance QTLs described to date are quantitative and inherited in a dominant manner. The next challenge is to incorporate the resistance QTLs, either individually or by stacking, into elite brassica cultivars. This would protect against virus infection and lessen the selection pressure for mutations resulting in TuYV isolates capable of infecting current OSR varieties possessing *TuYR1* and future OSR and vegetable varieties possessing *TuYR2‐TuYR9*. Introgression of these new resistances into commercial oilseed rape and vegetable brassicas can be accelerated by marker‐assisted breeding, using the molecular markers that have been developed.

Rigorous testing of resistances against different TuYV isolates is important to identify sources which provide broad‐spectrum resistance to TuYV. Additionally, testing the durability of resistance at higher temperatures will be important to identify resistance sources that provide long‐term potential of reducing TuYV load in the face of global climate change. The identification of the major genes responsible for resistance to TuYV remains elusive. Future identification of the actual resistance genes should be a priority, as it will enable plant‐virus protein interaction studies and may facilitate the rapid identification of additional TuYV resistance genes and/or associated alleles. There may also be opportunities for genetically engineering TuYV resistance, enabling further prospects of enhancing the durability of resistance to TuYV in brassica crops in the face of resistance‐breaking mutations and a changing environment.
